# Differential response of cancer cells to HDAC inhibitors trichostatin A and depsipeptide

**DOI:** 10.1038/bjc.2011.532

**Published:** 2011-12-08

**Authors:** J Chang, D S Varghese, M C Gillam, M Peyton, B Modi, R L Schiltz, L Girard, E D Martinez

**Affiliations:** 1 Hamon Center for Therapeutic Oncology Research, UT Southwestern Medical Center, 6000 Harry Hines Boulevard, Dallas, TX 75390-8593, USA; 2 Laboratory of Receptor Biology and Gene Expression, NCI, NIH, Bethesda, MD 20892, USA; 3 Department of Pharmacology, UT Southwestern Medical Center, 6000 Harry Hines Boulevard, Dallas, TX 75390-8593, USA

**Keywords:** trichostatin A, depsipeptide, HDAC inhibitors, cancer cell viability, drug sensitivity

## Abstract

**Background:**

Over the last decade, several drugs that inhibit class I and/or class II histone deacetylases (HDACs) have been identified, including trichostatin A, the cyclic depsipeptide FR901228 and the antibiotic apicidin. These compounds have had immediate application in cancer research because of their ability to reactivate aberrantly silenced tumour suppressor genes and/or block tumour cell growth. Although a number of HDAC inhibitors are being evaluated in preclinical cancer models and in clinical trials, little is known about the differences in their specific mechanism of action and about the unique determinants of cancer cell sensitivity to each of these inhibitors.

**Methods:**

Using a combination of cell viability assays, HDAC enzyme activity measurements, western blots for histone modifications, microarray gene expression analysis and qRT–PCR, we have characterised differences in trichostatin A *vs* depsipeptide-induced phenotypes in lung cancer, breast cancer and skin cancer cells and in normal cells and have then expanded these studies to other HDAC inhibitors.

**Results:**

Cell viability profiles across panels of lung cancer, breast cancer and melanoma cell lines showed distinct sensitivities to the pan-inhibitor TSA compared with the class 1 selective inhibitor depsipeptide. In several instances, the cell lines most sensitive to one inhibitor were most resistant to the other inhibitor, demonstrating these drugs act on at least some non-overlapping cellular targets. These differences were not explained by the HDAC selectivity of these inhibitors alone since apicidin, which is a class 1 selective compound similar to depsipeptide, also showed a unique drug sensitivity profile of its own. TSA had greater specificity for cancer *vs* normal cells compared with other HDAC inhibitors. In addition, at concentrations that blocked cancer cell viability, TSA effectively inhibited purified recombinant HDACs 1, 2 and 5 and moderately inhibited HDAC8, while depsipeptide did not inhibit the activity of purified HDACs *in vitro* but did in cellular extracts, suggesting a potentially indirect action of this drug. Although both depsipeptide and TSA increased levels of histone acetylation in cancer cells, only depsipeptide decreased global levels of transcriptionally repressive histone methylation marks. Analysis of gene expression profiles of an isogenic cell line pair that showed discrepant sensitivity to depsipeptide, suggested that resistance to this inhibitor may be mediated by increased expression of multidrug resistance genes triggered by exposure to chemotherapy as was confirmed by verapamil studies.

**Conclusion:**

Although generally thought to have similar activities, the HDAC modulators trichostatin A and depsipeptide demonstrated distinct phenotypes in the inhibition of cancer cell viability and of HDAC activity, in their selectivity for cancer *vs* normal cells, and in their effects on histone modifications. These differences in mode of action may bear on the future therapeutic and research application of these inhibitors.

Over the last decade, histone deacetylases (HDACs) have been identified as *bona fide* molecular targets for the treatment of various disorders including cancer. HDACs have pivotal roles in the regulation of gene expression, forming complexes with DNA binding proteins and thereby affecting histone acetylation and chromatin accessibility at promoter regions (Fischle *et al*, 2001; Ekwall, 2005; Clapier and Cairns, 2009; Witt *et al*, 2009). These enzymes also have non-histone substrates, such as transcription factors and structural proteins whose biological activity is partly regulated by acetylation (Di Gennaro *et al*, 2004; Chen *et al*, 2005; Zhao *et al*, 2006; Sadoul *et al*, 2008). Because deregulation of HDAC function has been found in cancer and other pathologies, inhibitors of HDACs have gained importance as potential therapeutics and as tools for chemical genomics investigations (Shames *et al*, 2006; Zhong *et al*, 2007; Marshall *et al*, 2009).

The HDAC family comprises four classes of deacetylases, grouped by their homology, their expression patterns and subcellular localisation, the cofactors required for their activity, and their substrates. Class 1, 2 and 4 HDACs share histones as substrates and utilise zinc for catalytic activity. Inhibitors of these enzymes contain zinc-chelating groups that disrupt enzyme/metal coordination at the active site, thus blocking catalytic activity. Among the well-established HDAC inhibitors, trichostatin A represents the hydroxamic acids which show general inhibition of class 1, 2 and 4 HDACs with nanomolar potency (Schultz *et al*, 2004; Bieliauskas and Pflum, 2008). Other inhibitors include the short chain fatty acid butyrate, the antibiotic apicidin, and the cyclic peptide FR901228 commonly known as depsipeptide. Depsipeptide has been shown to have selectivity for class 1 HDACs and alters histone modifications in cells at low nanomolar doses, although IC_50_ (50% inhibitory concentration) values for HDAC inhibition *in vitro* are reportedly significantly higher (Schrump *et al*, 2002; Bieliauskas and Pflum, 2008; Itoh *et al*, 2008).

These compounds have initially shown promising therapeutic potential in human cancer studies (McLaughlin and La Thangue, 2004; Villar-Garea and Esteller, 2004; Ryan *et al*, 2005; Lin *et al*, 2006; Riester *et al*, 2007; Jones and Steinkuhler, 2008; Prince *et al*, 2009; Rocca *et al*, 2009; Takai and Narahara, 2010); yet, the relationship between their HDAC inhibitory activity and their ability to control cancer growth is not clear. In addition, certain sub-populations of patients, benefit little or not at all and side effects range considerably (Schrump *et al*, 2002; McLaughlin and La Thangue, 2004; Kelly and Marks, 2005; Bolden *et al*, 2006; Stimson and La Thangue, 2009). At the molecular level, these differences can be partly explained by the fact that tumours express the various HDACs to different extents, and that current inhibitors are generally non-specific, targeting more than one HDAC enzyme and affecting non-histone as well as histone targets. Defining differences in HDAC inhibitor-induced phenotypes across inhibitor structural subgroups and understanding the molecular underpinnings that give rise to these differences is thus an important aspect of the preclinical development of this drug class. Although a number of groups have studied individual HDAC inhibitors and their effects on cell growth, these reports were not comparative across inhibitors and included only a few cell lines (Vigushin *et al*, 2001; Facchetti *et al*, 2004; Klisovic *et al*, 2005; Lee *et al*, 2006; Mukhopadhyay *et al*, 2006; Cantor *et al*, 2007; Miyanaga *et al*, 2008; Zhang *et al*, 2008).

Here, we profiled the sensitivity of lung cancer, breast cancer and skin cancer cell line panels in response to pan *vs* class 1 selective HDAC inhibitors and ranked cells according to their IC_50_ values. We also characterised the selectivity of these compounds by measuring their effects on normal human bronchial epithelial cells, human mammary epithelial cells and primary melanocytes and analysed their ability to block HDAC activity in purified systems and in cellular extracts at concentrations that impacted cancer cell viability. Furthermore, we identified differences in drug-induced phenotypes with respect to histone modifications and molecular determinants of sensitivity. Altogether, these findings may bear on the future use of these inhibitors and their analogues in personalised medicine applications.

## Materials and methods

### Cell culture

All human cancer cell lines were maintained in RPMI media supplemented with 5% (lung and breast cancer cells) or 10% (melanoma cells) fetal bovine serum. Human bronchial epithelial cells immortalised with cdk4 and telomerase were cultured in KSFM media supplemented with EGF and pituitary extract, as described (Ramirez *et al*, 2004). Human mammary epithelial cells immortalised with telomerase were maintained in complete MEGM media from Cambrex (East Rutherford, NJ, USA). Primary melanocytes were purchased from Cascade Biologics (Portland, OR, USA) and were cultured in Medium 254 with PMA-free human melanocyte growth supplement-2 (also from Cascade Biologics). TSA and apicidin were purchased from Alexis Biochemicals (Enzo Life Sciences, Farmington, NY, USA) and depsipeptide was the kind gift of Dr David Schrump.

### Cell viability assays and IC_50_ calculations

Cells were plated at 1500–3000 cells per well in 96-well plates and treated the next day with increasing doses of TSA, depsipeptide or apicidin over 4 days and their viability assessed by standard MTS assays using Promega's Cell Titer reagents (Promega, Madison, WI, USA) according to the manufacturer's protocols. Absorbance at 490 and 650 nm (reference wavelength) was measured by a Spectra Max (Molecular Devices, Sunnyvale, CA, USA) or a FlouroStar Omega (BMG Biosciences, Ortenberg, Germany) plate reader. Data were normalised to the untreated controls (100% viability). Each cell line was tested in 1–5 independent assays, each containing 4–8 replicates. IC_50_ values were calculated using DIVISA, a high-throughput software, developed in-house (manuscript in preparation), for storing and analysing drug sensitivity assays. Dose–response curves were plotted using a non-linear regression model and IC_50_s were determined from the fitted curves. Average IC_50_ values derived from 1 to 5 independent assays, each containing 4–8 replicates are shown in Tables 1, 2 and 3.

### HDAC assays

Cell extracts or purified recombinant HDAC enzymes were used in standard HDAC activity assays using Millipore's HDAC Fluorometric Assay Kit (Millipore, Billerica, MA, USA), according to the manufacturer's protocol. A FluoroStar Omega plate reader (BMG Biosciences) was used for fluorescent detection. Purified recombinant active HDACs 1, 5 and 8 were purchased from Millipore/Upstate. HDAC2 was purified as described below.

### Plasmid constructs for recombinant HDAC2 production

Two SDS–PAGE purified oligonucleotides (Eurofins, London, UK) encoding a carboxy terminal FLAG tag (DYKDDDDK) followed by a stop codon were phosphorylated with T4 polynucleotide kinase (NEB), annealed together and ligated into *Xho*I and *Hind*III digested pFastbBac1 (Invitrogen, Carlsbad, CA, USA) to generate pFastBac1-C-FLAG. The sequences of the oligonucleotides are 5′-TCGAGGACTACAAGGACGACGATGACAAATGA-3′ and 5′-AGCTTCATTTGTCATCGTCGTCCTTGTAGTCC-3′. The murine HDAC2 open reading frame was amplified by PCR from the plasmid pME18S-HDAC2 (a generous gift form Robert Eisenman). The sequences of the PCR amplification oligonucleotides (Eurofins) are 5′-GATCGGATCCATGGCGTACAGTCAAGGAGGCGGCAAG-3′ and 5′-GGATCTCGAGAGGGTTGCTGAGTTGTTCTGACTTG-3′. The resulting PCR product was digested with *Bam*HI (NEB) and *Xho*I (NEB) and ligated into *Bam*HI and *Xho*I digested pFastBac1-C-FLAG. The resulting plasmid is pFastBac1-mHDAC2-C-FLAG.

### Production of recombinant baculovirus and FLAG affinity purification of HDAC2

pFastBac1-mHDAC2-C-FLAG was used to produce recombinant baculovirus using the Bac-to-Bac Baculovirus Expression System (Invitrogen) according to the manufacturer's protocol. Recombinant murine HDAC2 baculovirus at an MOI of 10 was used to infect five T162 monolayer flasks seeded with 4 × 10^7^ Sf9 cells per flask in Sf900-II-SFM (Invitrogen) serum-free media. Infected cells were harvested 48 h post infection and processed for FLAG affinity purification as previously described in Supplementary Data (Qiu *et al*, 2006).

### Western blot analysis

Exponentially growing cells were treated with the appropriate compounds and harvested 4 or 24 h later in a standard nuclear lysis buffer and whole cell lysates were quantified. Equal amounts of total protein were loaded onto 4–12% Bis-Tris SDS gels and subjected to electrophoresis. After transferring onto nitrocellulose membranes, blots were probed for acetylated tubulin (Sigma, St Louis, MO, USA; T6793), acetylated histone 3 (Millipore/Upstate #06-599), acetylated histone 4 (Millipore/Upstate #07-329) or trimethylated histone 3 at lysine 9 (Millipore/Upstate #07-523). Actin (Santa Cruz, Santa Cruz, CA, USA; #1616) and HDAC1 (Affinity Bioreagents, Rockford, IL, USA; #PA1-860) were probed as loading controls. Quantification of bands in films was performed using two independent methods, which yielded similar results, Image J (NIH public software) and Quantity one (Bio-Rad, Hercules, CA, USA). The average of the two methods±s.e.m. is shown as specified in figure legends. For the MDR western analysis, a primary antibody against P-glycoprotein from Calbiochem (Darmstadt, Germany) was used according to the manufacturer's recommendations and HSP90 (Santa Cruz) was used as a loading control.

### Microarray gene expression profiles

Exponentially growing H1993 and H2073 cells were pelleted and RNA was extracted using the RNeasy kit (Qiagen, Valencia, CA, USA) according to the manufacturer's protocol. RNA quality was evaluated with the Experion gel system (Bio-Rad). Labelled RNA was hybridised to Affymetrix (Cleveland, OH, USA) HG-U133-Plus2 microarray chips according to the manufacturer's protocol and scanned using the Affymetrix GeneChip Scanner 2500. After pre-processing using the mas5 algorithm (Bioconductor, Affymetrix), gene expression changes were analysed using the Matrix 1.4 software package developed in-house. Selected genes showing differential expression levels greater than four-fold were further investigated by qRT–PCR and functional relationships were analysed with the Ingenuity Pathway gene annotation software (Ingenuity Systems, Redwood City, CA, USA).

### Quantitative real-time PCR

Exponentially growing cells were processed for RNA extraction using RNeasy (Qiagen). The extracted RNA was quantified, DNAse treated and reverse transcribed. The resulting cDNA was amplified in SybrGreen real-time quantitative PCR assays using validated primers specific for the genes of interest (sequences are given in Supplementary Table 2). Reactions were performed on an ABI Prism 7900HT (Life Technologies, Carlsbad, CA, USA), with an initial 2 min pre-incubation at 50°C, followed by 10 min at 95°C and then 40 cycles of 95°C for 15 s and 60°C for 1 min. Cyclophilin and 18S ribosomal RNA were used as references. Data were analysed following the ddCt method using a calibrator sample. Reactions were run in triplicate and error bars represent experimental error.

## Results

### Cancer cell sensitivity to TSA and depsipeptide show distinct profiles

**Figure 1:**
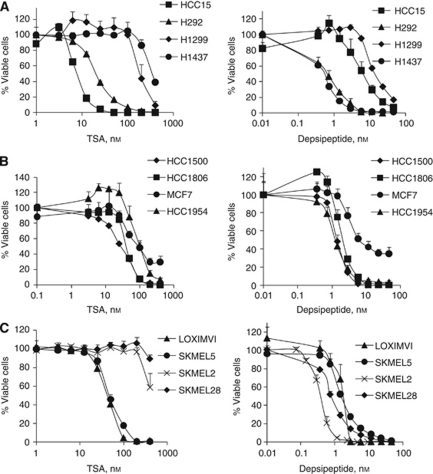
Human cancer cell lines show distinct sensitivities to HDAC inhibitors TSA and depsipeptide. The viability of human non-small lung cancer lines (**A**), breast cancer cells (**B**) and melanoma cells (**C**) was assessed after 4 days exposure to increasing concentrations of TSA (left panels) or depsipeptide (right panels) using standard MTS assays. See Supplementary Figure 1 for further cell line comparisons.

Our goals were to profile the sensitivity of cancer cells to the HDAC inhibitors trichostatin A and depsipeptide, to compare their relative potency and specificity across panels of lung cancer, breast cancer and melanoma cell lines, and to define phenotypes unique to each inhibitor. To assess whether human non-small cell lung cancer cells responded differentially to the pan-inhibitor TSA *vs* the class 1 selective inhibitor depsipeptide, we performed MTS cell viability assays for each drug on a panel of lines (Figure 1A; Supplementary Figure 1). IC_50_ measurements revealed that although some cell lines such as H292 and H1299 shared similar relative sensitivity to these two inhibitors (H292 is sensitive to both TSA and depsipeptide and H1299 is on the resistant end of both drug profiles, as shown in Figure 1A and Table 1), others showed opposite drug phenotypes being preferentially responsive to TSA and relatively resistant to depsipeptide or *vice versa*. HCC15, for example, demonstrated strong sensitivity to TSA, yet was among the most resistant lines to depsipeptide. Analogously, H1437 was strongly resistant to TSA treatment but was sensitive to depsipeptide. Similar differences across cell lines were also observed using other measures of cell viability (Supplementary Figures 2A and B).

Next, we wanted to evaluate if the distinct relative sensitivities to TSA *vs* depsipeptide of these lung cancer cell lines would generally hold for other pan *vs* class 1 selective inhibitors. Indeed, HCC15 cells were more sensitive to the pan-inhibitor Scriptaid compared with H1437 cells (Supplementary Figure 2C), following a TSA-like pattern. Similarly, MS-275, a class 1 selective HDAC inhibitor, exhibited a depsipeptide-like profile being more potent against H1437 cells than HCC15 cells (Supplementary Figure 2D). Thus, also in this case the relative sensitivity of H1437 and HCC15 cells to HDAC inhibitors of differential specificity followed opposite patterns.

To begin to understand cellular mechanisms that may contribute to the relative sensitivity of lung cancer cells to TSA or depsipeptide, we performed qRT–PCR analysis measuring HDAC levels in a subset of lines. Interestingly, we found that HDAC2 was markedly upregulated in H1299 cells, which showed general resistance to both TSA and depsipeptide, compared with H292 cells, which are sensitive to both inhibitors (Supplementary Figure 3A). Other class 1 HDACs also showed a slight trend of upregulation in H1299. In contrast, when comparing levels of HDACs in cells with distinct sensitivities to TSA *vs* depsipeptide, such as H1437 to HCC15, we saw no clear differences in class 1 HDAC levels (Supplementary Figure 3B). Instead, we did observe an upregulation of class 2 HDACs in HCC15 *vs* H1437 (Supplementary Figure 3C), suggesting the relative resistance of HCC15 to depsipeptide may in part be related to the uninhibited and redundant action of the upregulated class 2 HDACs.

Differences in relative sensitivity to HDAC inhibitors were also observed in human breast cancer lines. As can be seen in Figure 1B, MCF7 cells responded differently to TSA than to depsipeptide: while they showed intermediate sensitivity to TSA treatment, MCF7 cells were relatively resistant to depsipeptide and exhibited a 30–40% surviving population. In contrast, HCC1954 cells were the most resistant tested breast cancer line to TSA, but the most sensitive line to depsipeptide (Figure 1B; Table 2). Differential patterns were also seen in melanoma cells, which showed distinct sensitivities to the two inhibitors as well (Figure 1C; Table 2). Strikingly, SK-MEL2 and SK-MEL28 were the most sensitive cutaneous melanomas to depsipeptide inhibition but were unaffected by TSA treatment except at the highest dose. SK-MEL5 and LOXIMVI, on the other hand, showed low sensitivity to depsipeptide but high sensitivity to TSA.

**Figure 2:**
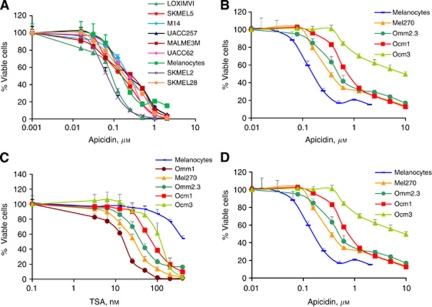
Unique cell viability profiles of apicidin, TSA and depsipeptide across cutaneous and uveal melanomas. Cutaneous (**A**) and uveal melanoma cells (**B**) were treated with increasing doses of apicidin and cell viability was measured by MTS assays after 4 days. The sensitivity of uveal melanoma cells to TSA (**C**) and depsipeptide (**D**) was also measured and compared with the response of primary melanocytes.

To further evaluate the behaviour of cells in response to HDAC inhibitors, we measured the effects of a third, structurally distinct compound, apicidin, on the melanoma cell panel and expanded the study to include uveal melanomas (Ren *et al*, 2004). Apicidin, which like depsipeptide has some selectivity for class 1 HDACs (Bieliauskas and Pflum, 2008), did not generally mimic the drug sensitivity profiles of either depsipeptide or TSA, showing a narrower range of activity with a smaller distribution of IC_50_ values across the non-uveal melanoma cell line panel (compare Figure 2A with Figure 1C). Nevertheless, SK-MEL2 was on the sensitive side of the apicidin spectrum in a depsipeptide-like manner but LOXIMVI was also sensitive to apicidin, following the TSA pattern. In uveal melanomas, TSA and apicidin clearly showed similar sensitivity profiles with IC_50_ values for Mel270<Omm2.3<Ocm1<Ocm3 for both compounds (Figure 2B and C). In contrast to Ocm3's relative resistance to TSA and apicidin, this cell line was sensitive to depsipeptide (Figure 2D), demonstrating that cells can respond differentially even to structurally distinct class 1 selective HDAC inhibitors. Taken together, these data suggest that indeed, each HDAC inhibitor structural class has a unique drug sensitivity profile indicative of differences in the mode of action of these compounds and in their interaction with cellular targets. As discussed below, the specificity of HDAC inhibitors for cancer *vs* normal cells also exhibited unique patterns across compounds and tissue types.

### TSA but not depsipeptide or apicidin shows general specificity for cancer *vs* normal cells

**Figure 3:**
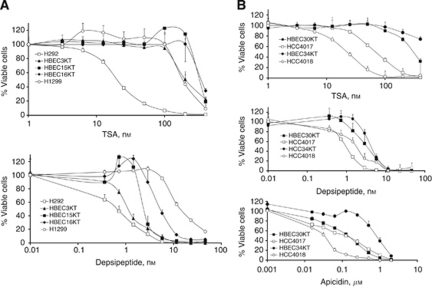
TSA shows greater specificity for cancer than depsipeptide and apicidin. (**A**) Immortalised human bronchial epithelial cells were treated with TSA (top) or depsipeptide (bottom) in a dose–response series and cell viability assessed 4 days later. H292, which is on the sensitive side of the TSA response and of the depsipeptide response is shown for comparison. H1299, a line on the resistant end of both the TSA and depsipeptide profiles is also shown. HBECs are resistant to TSA but show intermediate sensitivity to depsipeptide. (**B**) Two patient-matched pairs of human bronchial epithelial cells/human non-small lung cancer cells were treated with increasing concentrations of the three HDAC inhibitors. TSA showed the largest and most general selectivity for cancer *vs* normal cells. HCC4017 was derived from the same patient as HBEC30KT, and HCC4018 was derived from the same patient as HBEC34KT.

To determine if normal cells also showed a differential response to HDAC inhibitors and to measure the selectivity of these drugs for cancer cells *vs* normal cells, we performed cell viability assays on a series of immortalised human bronchial epithelial cells (HBECs) that are non-tumourigenic (Ramirez *et al*, 2004; Sato *et al*, 2006). TSA was cancer selective, showing higher IC_50_ values for HBECs than for most lung cancer cells (Figure 3A; Table 3). In contrast, depsipeptide lacked this specificity and blocked the viability of HBECs with similar or greater potency than of lung cancer cells (Figure 3A bottom panel and compare Table 1 values with Table 3 values). The greater ability of TSA *vs* depsipeptide to preferentially target human cancer *vs* mammary epithelial cells was also seen in breast cells (Table 2; Supplementary Figure 1B). On the other hand, primary melanocytes showed resistance to both TSA and depsipeptide compared with cutaneous melanomas, but melanocytes were as sensitive to apicidin as most melanoma cells. Compared with uveal melanomas, primary melanocytes showed greater resistance to TSA than even Ocm3 cells – which have a high IC_50_ for this drug – moderate resistance to depsipeptide and full sensitivity to apicidin (Tables 2 and 3). Thus, TSA is the most selective of these three HDAC inhibitors while apicidin generally lacks selectivity for cancer *vs* normal cells.

**Figure 4:**
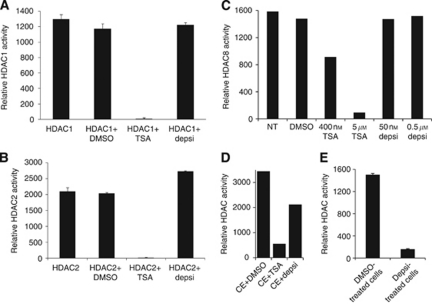
TSA inhibits HDAC activity *in vitro* at doses that block cancer cell growth. (**A**) The activity of purified HDAC1 was measured in the presence of 400 nM TSA or 50 nM depsipeptide, as indicated. (**B**) HDAC2 activity was quantified *in vitro* after treatment with vehicle, 400 nM TSA or 0.5 *μ*M depsipeptide. (**C**) HDAC8 activity was measured in the presence of the indicated drug treatments. (**D**) Cell extracts treated *in vitro* with 150 nM TSA or 20 nM depsipeptide showed decreased total HDAC activity. (**E**) Lysates from cells treated with 25 nM depsipeptide show a strongly reduced total HDAC activity. C127 cells which have good levels of HDACs were used for (**D**) and (**E**). Drug-treated Baf3 lysates gave equivalent results.

A comparison of TSA's effect on matched pairs of lung cancer and HBEC lines derived from the same patient further demonstrated its preferential selectivity for cancer cells. In two independent pairs, TSA showed greater potency against the lung cancer line than against the corresponding patient-matched HBEC line, with a difference in IC_50_ values within a pair ranging from 5- to ≫20-fold (Figure 3B; Table 3). Depsipeptide showed a slight selectivity of about three- to five-fold and apicidin was equally potent against one matched lung cancer/HBEC pair showing no selectivity, but was ∼25-fold specific for cancer in the second pair of lines (Figure 4C). These results confirm the hydroxamic acid TSA as the most cancer-specific HDAC inhibitor compared with the other compounds tested.

### TSA inhibits HDAC activity at concentrations that block cancer cell viability and depsipeptide lowers global histone methylation levels

To explore whether the function of HDACs was modulated by inhibitors at concentrations that block cancer cell viability, we performed enzyme activity assays in purified systems and in cell extracts. TSA's effective killing dose of 400 nM fully inhibited HDAC1 activity, while depsipeptide's effective killing dose of 50 nM produced no inhibition (Figure 4A). Similarly, HDAC2 enzymatic function was fully inhibited by 400 nM TSA, but depsipeptide did not decrease HDAC2 activity, which remained unaffected even in the presence of 10-fold higher concentrations of depsipeptide (Figure 4B). HDAC5 activity was similarly sensitive to TSA inhibition but not to depsipeptide (data not shown). As shown in Figure 4C, the most resistant enzyme was HDAC8, which was only partly inhibited by 400 nM TSA, a concentration that fully inhibits cancer cell viability. However, 5 *μ*M TSA fully eliminated its activity. Depsipeptide at either 50 nM or 0.5 *μ*M had no effect on HDAC8. Interestingly, total HDAC activity in cell extracts treated with inhibitors *in vitro* was blocked by TSA and was partly inhibited by depsipeptide (Figure 4D). Furthermore, lysates made from depsipeptide treated cells showed a strong inhibition of total HDAC activity compared with lysates made from DMSO-treated cells (Figure 4E), suggesting that depsipeptide requires activation by cellular enzymes and that it may exert at least some of its biological effects through mechanisms that affect HDAC activity indirectly in intact cells.

**Figure 5:**
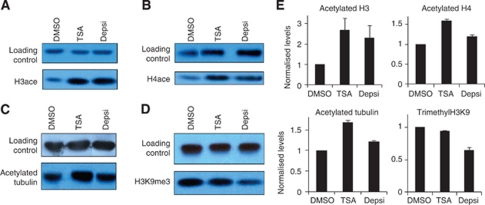
Depsipeptide causes a decrease in global H3K9 methylation levels concomitant with changes in histone acetylation. (**A**) Western blot analysis of non-small cell lung cancer H358 cells treated for 24 h with 200 nM TSA, 25 nM depsipeptide or vehicle showed increased levels of histone 3 acetylation in response to both inhibitors. (**B**) Cells treated as in (**A**) but for 4 h were assessed for global levels of histone 4 acetylation. (**C**) Acetylated tubulin levels in H358 cells treated for 24 h as in (**A**) increase in response to TSA. (**D**) H358 cells treated with 25 nM depsipeptide but not those treated with 200 nM TSA showed lowered levels of histone 3 lysine 9 trimethylation. Irrelevant bands were removed from gels for clarity of presentation. (**E**) Quantification of western analysis. The average quantification using two independent methods is shown, normalised to DMSO values. For acetylated tubulin two loading controls were run (actin, shown in **C** and HDAC1) giving similar results. The average of the two controls is shown. Error bars represent s.e.m.

Consistent with this inhibition of HDAC activity, we found that both TSA and depsipeptide had the ability to alter global histone modifications in lung cancer cells treated with these compounds. Western blot analysis of H358 cells showed that TSA as well as depsipeptide globally increased levels of histone 3 and histone 4 acetylation (Figures 5A, B and E). TSA also altered the amounts of acetylated tubulin, a known non-histone substrate of HDAC6 (Figures 5C and E). Given the ability of TSA and depsipeptide to increase histone acetylation and thus shift chromatin towards a state of higher transcriptional activity, we evaluated whether these acetylation effects would be accompanied by HDAC-independent changes in histone methylation at repressive marks. Remarkably, we found that depsipeptide treatment reproducibly decreased global levels of histone 3 trimethylated at lysine 9, while TSA had no effect on this repressive modification (Figures 5D and E). Thus, it appears that depsipeptide's mode of action involves mechanisms that both increase transcriptionally permissive marks on histones and that directly or indirectly decrease transcriptionally repressive marks, unlike TSA.

### Determinants of HDAC inhibitor sensitivity

**Figure 6:**
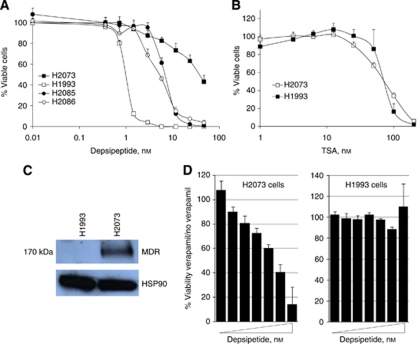
A primary/lymph node cell line pair shows distinct sensitivity to depsipeptide due to chemo-induced upregulation of MDR genes. (**A**) The sensitivity of two matched primary/lymph node cell line pairs in response to increasing amounts of depsipeptide was measured by MTS assays after 4 days of drug exposure. The H2085/H2086 pair showed almost identical dose–response curves, while the H1993 was remarkably more sensitivity to inhibition by depsipeptide than H2073. (**B**) The H2073/H1993 pair showed no differential sensitivity to TSA. (**C**) Western blot analysis confirmed upregulation of the ABCB1/MDR1 protein in the chemo-resistant H2073 line. (**D**) Inhibition of ABCB1/MDR1 by verapamil partly restores sensitivity to depsipeptide in H2073 cells without affecting H1993.

We next studied the drug responsiveness of matched pairs of cancer cell lines to begin to understand the determinants of cellular sensitivity to specific HDAC inhibitors. H2073 and H1993 are derived from the primary lung tumour and a lymph node metastatic lesion, respectively, of the same patient. H1993 was established before and H2073 after the patient had undergone chemotherapy (Phelps *et al*, 1996). Strikingly, although H2073 and H1993 show very similar sensitivity to TSA, H1993 is highly responsive to depsipeptide while H2073 is >25-fold more resistant to this drug (Figure 6A). A second pair of primary/lymph node cell lines showed almost identical sensitivity to depsipeptide (Figure 6A, H2085/H2086), demonstrating the differential sensitivity to depsipeptide is particular to the H2073/H1993 pair and not to primary *vs* metastatic cells. We reasoned, therefore, that studying what distinguishes H2073 from H1993 could help define the sources of phenotypic differences in the response to HDAC inhibitors and give insights into resistance to targeted therapy, which may develop after exposure to chemotherapy, as in the case of H2073.

Microarray analysis comparing gene expression profiles of H2073 *vs* H1993 uncovered groups of genes with distinguishable levels of expression across the cell line pair (Supplementary Table 1). H1993 showed higher expression than H2073 of certain histone variants and chromatin factors, targets that may specifically contribute to sensitivity to depsipeptide and may be downregulated by chemotherapy (Supplementary Table 1; Supplementary Figure 4). For example, H1993 expresses about five-fold higher levels of JMJD2B, a Jumonji enzyme that demethylates trimethylated H3 at K9, than H2073. Yet, there are no changes in the expression of the functionally antagonistic histone methylases across this cell line pair (not shown). Given that depsipeptide but not TSA action involves decreases in H3K9 trimethylation levels, it can be speculated that the higher levels of JMJD2B in H1993 would facilitate depsipeptide action. Among the genes differentially expressed in H2073 compared with H1993, genes involved in glutathione metabolism were strongly upregulated as validated by qRT–PCR analysis (Supplementary Table 1; Supplementary Figure 4), suggesting that depsipeptide action but not TSA action is thwarted by glutathione detoxification pathways in H2073. This is interesting since although depsipeptide is thought to require reduction for its cellular activity (mediated by glutathione and other cellular reducers), the reduced drug is significantly less stable and chemotherapy-related ROS production can interfere with depsipeptide reduction (Furumai *et al*, 2002; Crabb *et al*, 2008; Itoh *et al*, 2008).

A second group of highly upregulated genes in H2073 were members of the multidrug resistance family, namely ABCB1 (MDR1), ABCC2 (MRP2) and ABCC6 (MRP6). Depsipeptide, but not TSA, has been reported to be a substrate of ABCB1/MDR1 (Peart *et al*, 2003). Thus, it is likely that depsipeptide effluxes from H2073 cells and that this contributes to their resistance. In agreement with this idea, we found high protein levels of ABCB1/MDR1 in H2073 cells compared with undetectable levels in H1993 cells (Figure 6C) and treatment of H2073 cells with the MDR1 inhibitor verapamil partly re-sensitised H2073 cells to depsipeptide while not affecting H1993 cells (Figure 6D). Taken together, these results suggest that chemotherapy-resistant residual disease will likely also be resistant to depsipeptide primarily due to the upregulation of MDR genes but may still respond to TSA.

## Discussion

By studying the drug sensitivity profiles of several HDAC inhibiors across panels of lung cancer, breast cancer and melanoma cells we have identified unique patterns of drug response specific for each inhibitor. Interestingly, the pan-inhibitor TSA showed greater specificity for cancer *vs* normal cells than the class 1 selective inhibitors depsipeptide and apicidin. TSA inhibited HDAC activity in purified systems *in vitro*, while depsipeptide's inhibition of HDACs at doses that blocked cancer cell viability required components of cell extracts. Consistent with this, we found that although both drugs increased global histone acetylation levels, only depsipeptide caused a concomitant decrease in global levels of the repressive histone 3 lysine 9 trimethylation mark, indicating that depsipeptide may indirectly affect other factors in addition to its HDAC inhibitory activity. Microarray gene expression analysis of an isogenic cell line pair, which showed identical sensitivity to TSA but discrepant sensitivity to depsipeptide, revealed that resistance to the cyclic peptide inhibitor may be mediated at least in part by chemotherapy-induced increases in multidrug resistance gene expression. Thus, in spite of targeting the same enzyme family, the various HDAC inhibitors studied here each demonstrated unique phenotypes that may impact the current clinical development of this drug class.

The divergent patterns of drug sensitivity of human cancer cells across HDAC inhibitors we observed allowed us to conclude that each inhibitor, including those that have selectivity for class 1 HDACs, must have activities beyond just HDAC inhibition, which contribute to overall drug effects such as cytotoxicity. In the case of depsipeptide, one such activity may be its ability to lower global levels of histone methylation as we observed at concentrations that blocked the viability of cancer cells. Consistent with this, Wu *et al* (2008) have reported decreased H3K9 methylation at promoters of genes upregulated by depsipeptide. The global changes in H3K9 methylation we see in response to depsipeptide but not TSA suggest that depsipeptide may potentially affect the activity or level of histone methylases or demethylases directly, as was shown in H719 cells for G9a and SUV39H1 (Wu *et al*, 2008). We did not observe any significant differences, however, in the basal levels of these methylases across cells with a range of depsipeptide sensitivities (data not shown).

In our analysis of drug sensitivity profiles, we found that cancer cell responsiveness to the HDAC inhibitors tested in this study was independent of the histological characteristics and the stage of the tumour from which the cell lines were derived. Likewise, we found no general correlation between drug sensitivity and the available mutational status of cells. Interestingly, however, one of the cell lines that showed resistance to TSA but was sensitive to depsipeptide, H1437, has been recently found to harbour a mutation in HDAC9 (Forbes *et al*, 2010), a class 2 HDAC. Whether this may be a feature that contributes to TSA resistance generally, remains open to investigation. It is tempting to speculate that molecular determinant of responsiveness may be uncovered by a more thorough analysis of the molecular features of the cell line panels used here, analogous to the discovery of the correlation between EGFR mutations and hypersensitivity to tyrosine kinase inhibitors (Lynch *et al*, 2004).

Surprisingly, we did see a large shift in responsiveness to depsipeptide, but not other HDAC inhibitors, in two cell lines, one derived from a lymph node lesion before chemotherapy treatment, the other derived from the primary tumour after exposure to etoposide and cisplatin. Comparison of this isogenic pair by microarray gene expression profiling indicated that higher levels of enzymes in the glutathione pathway corresponded to resistance to depsipeptide seen in the line derived after chemotherapy. Xiao *et al* (2003) reported the presence of various glutathione-depsipeptide conjugates in serum, and proposed that these may represent metabolites with altered activity. In addition, depsipeptide's disulphide bond is subject to reduction and this appears to require cytosolic activities and to increase the potency of depsipeptide and its analogues (Xiao *et al*, 2003; Crabb *et al*, 2008), yet decreasing the active drug's stability. The glutathione pathway enzymes upregulated in H2073 may potentially affect this reduction and/or increase depsipeptide conjugation to reduced glutathione (through the action of GSTP1, GSTA4 and GSTM3, for example), keeping the cyclic peptide in a less stable form. The connection between drug potency and the status of the disulphide bond within depsipeptide may also help explain the lack of inhibition of HDACs in purified systems at concentrations that had clear biological effects in cells and lysates. Whether sensitivity to depsipeptide may be compromised by prior exposure to chemotherapy in clinical settings remains an open possibility, supported by our data, which must be taken into consideration for future trials.

Despite the greater general potency of depsipeptide compared with TSA or apicidin, the specificity profile of this drug was inferior to that of TSA. Thus, although less selective as an HDAC inhibitor, TSA was the most specific anticancer agent, preferentially targeting cancer *vs* normal cells. A TSA analogue, SAHA/vorinostat, has been approved for clinical use by the FDA against cutaneous T-cell lymphoma (Duvic and Vu, 2007) with a toxicology and side effect profile that meets standards. To date, therapeutic use of depsipeptide or apicidin analogues or other HDAC inhibitors has not been approved, although clinical trials are ongoing (Sandor *et al*, 2002; Ryan *et al*, 2005; Arce *et al*, 2006; Candelaria *et al*, 2007; Prince *et al*, 2009; Rocca *et al*, 2009). Our study suggests that at least in the preclinical setting, TSA-related compounds may offer the better blend of effectiveness and specificity for lung and breast cancer and melanomas. This may potentially also hold in the setting of combination therapy. A recent study by Frenkel, Gazdar and colleagues concluded that depsipeptide co-administered with EGFR inhibitors gave a substantial advantage over EGFR inhibitors alone in xenograft models of lung cancer (Zhang *et al*, 2009). Parallel studies comparing several HDAC inhibitors in combination with standard chemotherapies or targeted therapies will be needed to identify the most effective and safe combinations. Since vorinostat already has approval status, this would be a rational first choice of HDAC inhibitor, as several cancer centres with currently open trials have already realised.

## Figures and Tables

**Table 1:** IC_50_ values and sensitivity ranking of lung cancer cells

**NSCLC**	**TSA IC_50_**	**TSA rank**	**Depsipeptide IC_50_**	**Depsipeptide rank**
HCC15	7.9 nM	1	6.4 nM	12
H292	22.5	2	0.7	2
H2009	38.3	3	0.9	3
A549	39.7	4	2.0	7
H358	40	5	1.1	4
H1395	41	6	5.3	11
H820	55	7	4.0	10
H2073	63.7	8	37.1	15
H1993	93.5	9	1.2	5
H1355	107	10	1.8	6
HCC366	147	11	3.1	8
H1299	150	12	12.1	14
H1437	191	13	0.5	1
H2086	275	14	3.8	9
H2085	390	15	6.9	13

Abbreviations: IC_50_=50% inhibitory concentration; NSCLC=non-small cell lung cancer cell; TSA=trichostatin A.

**Table 2:** IC_50_ values and sensitivity ranking of breast cancer and melanoma cells

**BCa line**	**TSA IC_50_**	**TSA rank**	**Depsipeptide IC_50_**	**Depsipeptide rank**
HCC1500	29 nM	1	1.5 nM	4
HCC1806	41	2	1.8	5
348T^a^	81	3	1.3	2
MCF-7	90	4	4.2	7
HCC1954	91	5	1.5	3
MCF-10	96	6	2.4	6
1585T^a^	124	7	1.3	1
				
**Melanoma**	**TSA IC_50_**	**TSA rank**	**Depsipeptide IC_50_**	**Depsipeptide rank**
LOXIMVI	57 nM	1	1.2 nM	5
SKMEL5	66	2	1.5	6
M14	98	3	2.4	8
UACC257	222	4	1.6	7
UACC62	238	5	0.9	4
MALME3M	240	6	0.7	2
SKMEL2	336	7	0.4	1
SKMEL28	400	8	0.8	3
				
**Uveal Melanoma**	**TSA IC_50_**	**Depsipeptide IC_50_**		**Apicidin IC_50_**
MEL270	68 nM	1.8 nM		0.65 *μ*M
OCM1	188	5.5		0.7
OCM3	220	1.5		4
OMM2.3	81	2.7		0.4
				

Abbreviations: IC_50_=50% inhibitory concentration; TSA=trichostatin A.

a Immortalised normal human mammary epithelial cells.

**Table 3:** IC_50_ values for human bronchial epithelial cells, patient matched normal/lung cancer lines and melanocytes

**Cell line**	**TSA IC_50_, nM**		**Depsipeptide IC_50_, nM**
HBEC3KT	318		1
HBEC15KT	295		2.5
HBEC16KT	260		4.9
			
**Cell line**	**TSA IC_50_, nM**	**Depsipeptide IC_50_, nM**	**Apicidin IC_50_, *μ*M**
HBEC30KT	285	0.9	0.09
HCC4017	73	6.5	0.12
HBEC34KT	346	1.3	0.79
HCC4018	23	4.2	0.03
Melanocytes	284	0.9	0.2

Abbreviations: IC_50_=50% inhibitory concentration; TSA=trichostatin A.
