# Supplementary material for: Differential response of cancer cells to HDAC inhibitors trichostatin A and depsipeptide
Source: Br J Cancer. 2011 Dec 8;106(1):116–25. doi: 10.1038/bjc.2011.532 (PMC3251870; doi:10.1038/bjc.2011.532)
Supplement: Supplementary Material [file bjc2011532x2.pdf]

Supplementary Material Chang et al

This supplementary material contains:

-Supplementary Figure Legends

-Supplementary Figures 1-4

-Supplementary Table 2

Please see separate excel file for Supplementary Table 1.

Supplementary Figure Legends

Supplementary Figure 1: Expanded panel of cell lines showing distinct relative sensitivities to TSA vs. depsipeptide. Lung cancer cell lines not depicted in the main Figure 1A are shown in A) and an expanded set of breast cancer cells and HMES and melanoma cells and melanocytes in C).

Supplementary Figure 2: Differential response to HDAC inhibitors is a general phenomenon. A) Cell viability measured by ATP levels in response to TSA also shows the relative sensitivity of HCC15 compared to H1299. B) Similarly, H1437 shows greater sensitivity to depsipeptide than H1299 also by ATP measurements. C) Sensitivity to scriptaid, a pan-inhibitor, mimics TSA while D) MS-275 sensitivity mimics depsipeptide in the HCC15/H1437 comparison.

Supplementary Figure 3: HDAC expression levels may contribute to drug sensitivity. A) Members of class 1 HDACs are upregulated in lung cancer lines which are sensitive to both TSA and depsipeptide compared to drug resistant lines. B) HDAC 2 levels are similar in the cell line pair showing most differential responses to TSA vs. depsipeptide while class 2 HDACs are upregulated in the depsipeptide resistant line HCC15 as shown in C), as measured by qRTPCR.

Supplementary Figure 4: Validation of microarray gene expression differences in the H1993/H2073 cell line pair confirmed overexpression of glutathione metabolism genes in H2073 (shown in A) and downregulation of chromatin interacting proteins (B).

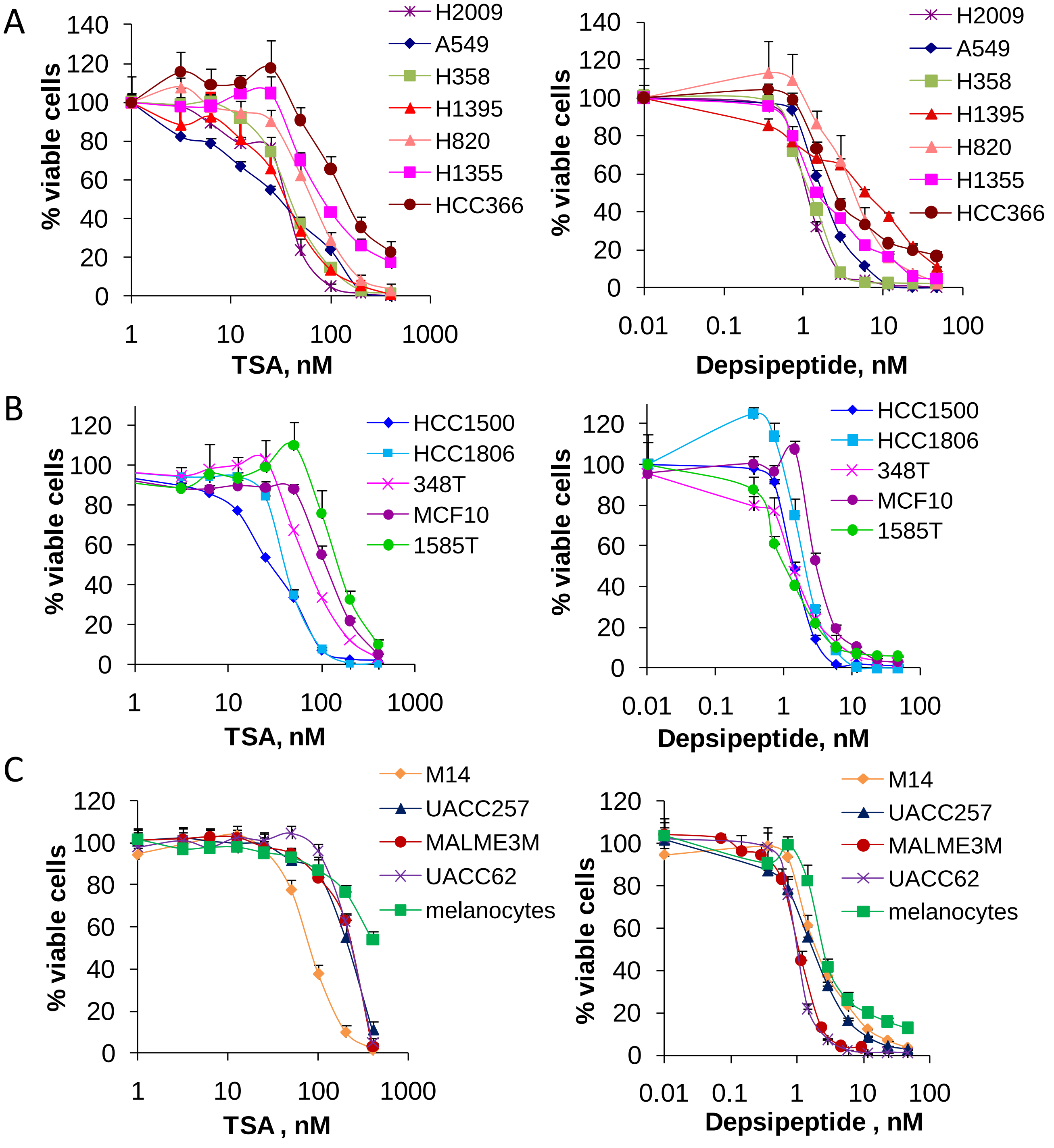

Supplementary Figure 1

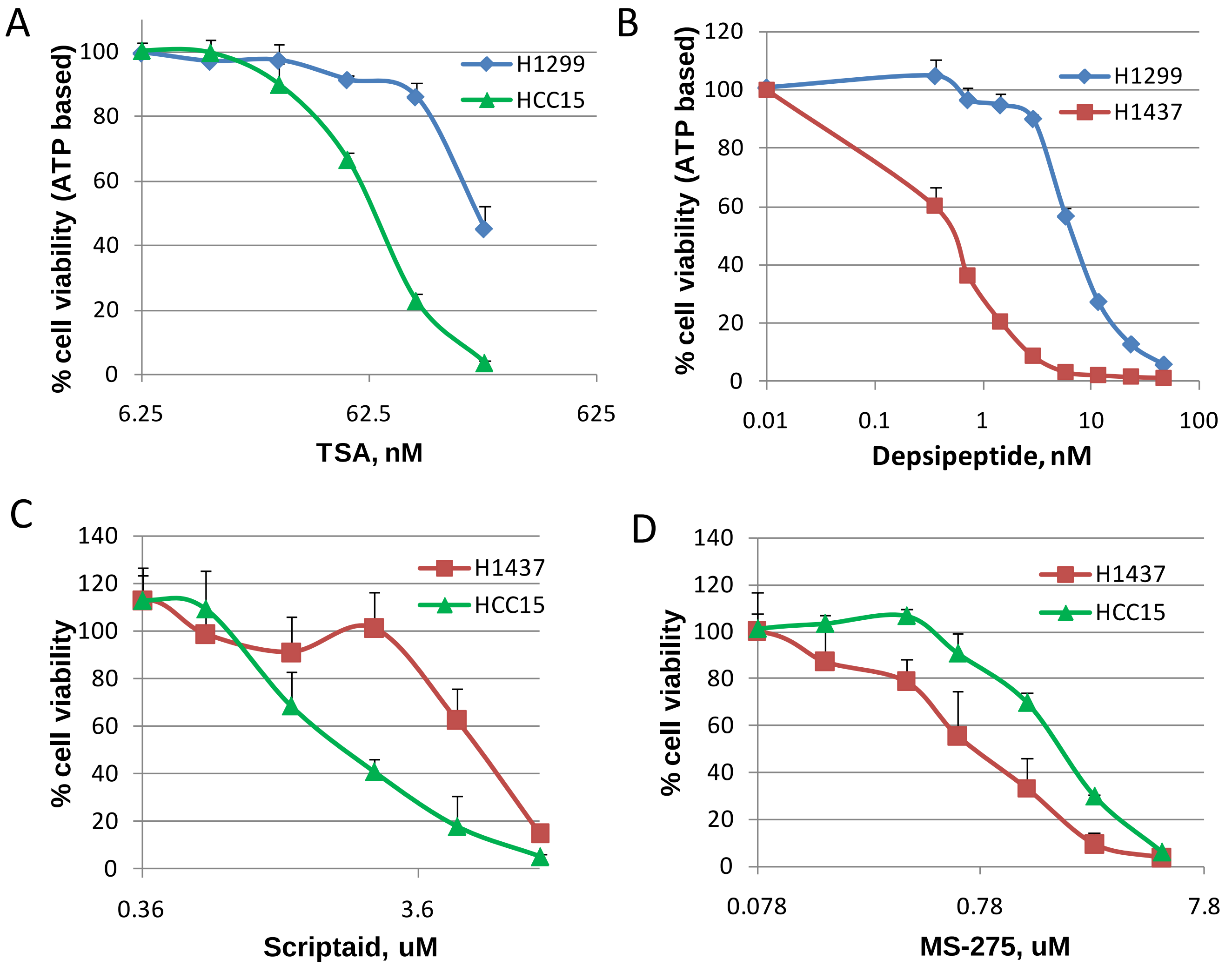

Supplementary Figure 2

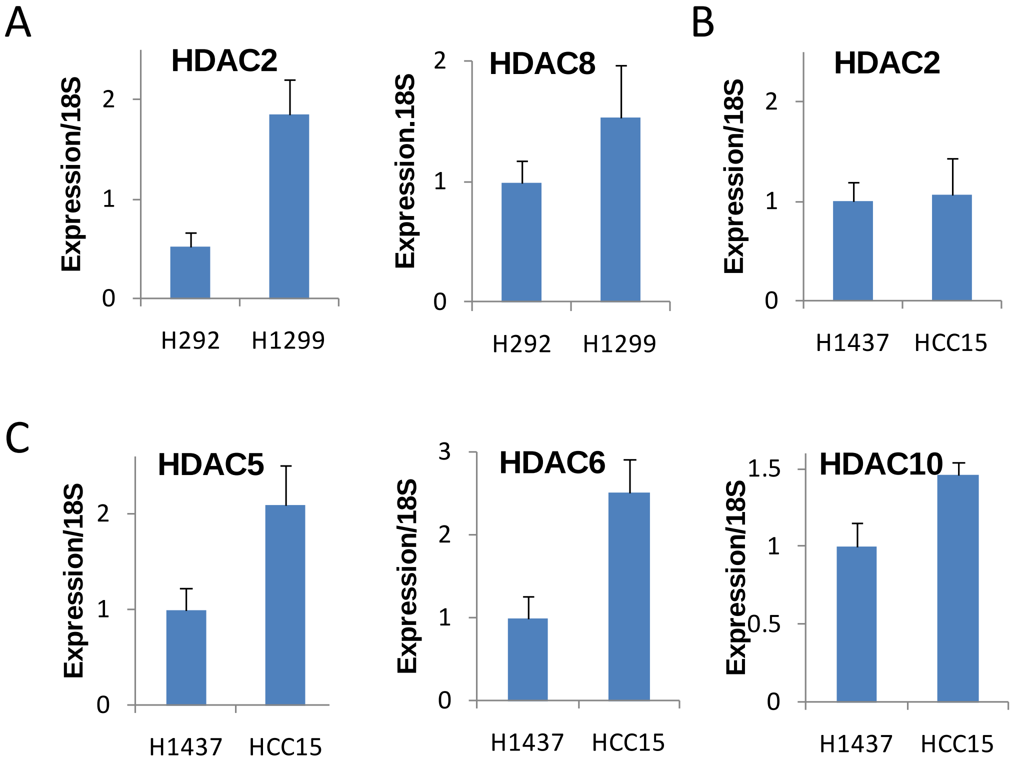

Supplementary Figure 3

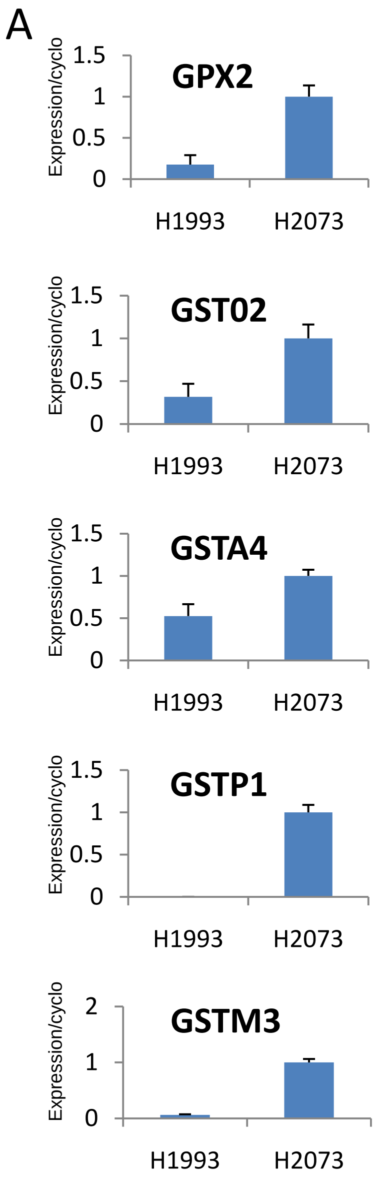

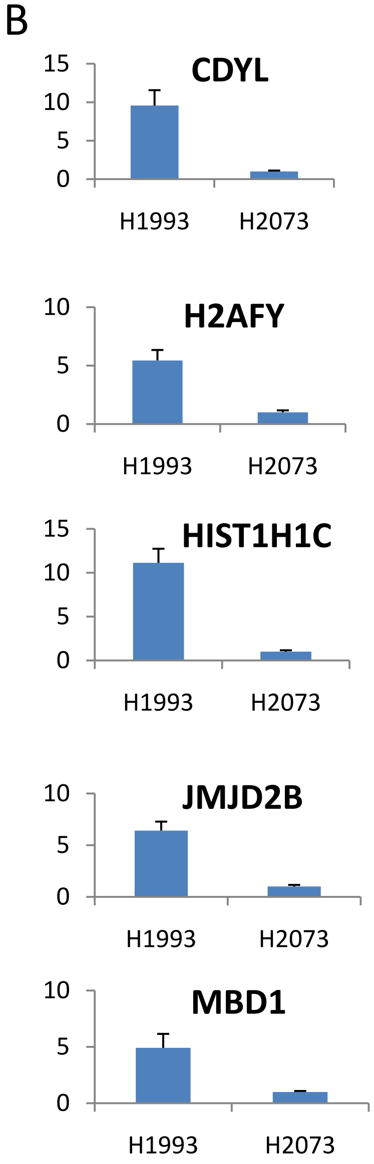

Supplementary Figure 4

Supplementary Table 2: Sequences of validated qRTPCR primer pairs used in this study

**Gene Symbol**	**Primer sequences used**
hdac2	F: 5'TCAAGGAGGCGGCAAAAA3'<br>R: 5'TGCGGATTCTATGAGGCTTCA3'
hdac5	F: 5'CCATTGGAGACGTGGAGTACCT3'<br>R: 5'GCGGAGACTAGGACCACATCA3'
hdac6	F: 5'GGAATGGCATGGCCATCATTAG3'<br>R: 5'CGTGGTTGAACATGCAATAGC3'
hdac8	F: 5'GACCGTGTCCCTGCACAAA3'<br>R: 5'CAACATCAGACACGTCACCTGTT3'
hdac10	F: 5'GCTTCACTGTCAACCTGCCC3'<br>R: 5'AGTCAGCGTTTCCCATCCC3'
gpx2	F: 5'TGGCTTCCCTTGCAACCA3'<br>R: 5'TCCTCATTCTGACAGTTCTCCTGAT3'
gsto2	F: 5'CGTCCTCAAGGCCAAAGACA3'<br>R: 5'TCTCAGGTTAATGTTGACCACTTCA3'
gsta4	F: 5'TGCGGCTCCAGGCCT3'<br>R: 5'AGCTTGGGCCTTGCTGC3'
gstp1	F: 5'CGCAGTCTTCGCCACCA3'<br>R: 5'CGAACTGGGAAATAGACCACG3'
gstm3	F: 5'AGAAACGGTACACGTGCGG3'<br>R: 5'GCCATTGGCTTCGATCATAGTC3'
cdyl	F: 5'CACTAAAATGGCAGAAGCTATCAGAA3'<br>R: 5'GGGCCATTGACTGCTACAATAAT3'
h2afy	F: 5'GTGGCCAATGATGAAGAGCTG3'<br>R: 5'GGCTATGGTGACTCCTTTTAGCAG3'
hist1h1c	F: 5'TTTGCCACTTGTACCCGAGTT<br>R: 5'GGAGCAGTCTCGGACATGTTG3'
jmjd2b	F: 5'GGCCTCTTCACGCAGTACAATAT3'<br>R: 5'CCAGTATTTGCGTTCAAGGTCAT3'
mbd1	F: 5'GCGCCGCGAAGTCTTTC3'<br>R: 5'TCGAGTCAGCTCAACTTTGCTT3'
